# Risk factors of major bleeding associated with direct oral anticoagulant treatment in patients with venous thromboembolism: a systematic review and meta-analysis

**DOI:** 10.3389/fmed.2026.1869818

**Published:** 2026-10-15

**Authors:** Hairong Zhan, Fuxin Ma, Jintuo Zhou, Jinhua Zhang

**Affiliations:** Department of Pharmacy, Fujian Maternity and Child Health Hospital College of Clinical Medicine for Obstetrics & Gynecology and Pediatrics, Fujian Medical University, Fuzhou, China

**Keywords:** direct oral anticoagulants, major bleeding, meta-analysis, risk factor, treatment

## Abstract

**Background:**

Direct oral anticoagulants (DOACs) are commonly prescribed for venous thromboembolism (VTE), but major bleeding (MB) remains an important complication of treatment. Despite their widespread use, systematic evaluations of prognostic factors associated with major bleeding during DOAC treatment remain scarce. To address this gap, we conducted a systematic review to identify prognostic factors associated with major bleeding during DOAC treatment in patients with VTE. This study seeks to enhance the safety and efficacy of anticoagulation therapy for VTE patients.

**Method and results:**

A systematic review and meta-analysis were conducted to search the PubMed and Web of Science databases for studies published before September 18, 2026. The review adhered to predefined inclusion and exclusion criteria to identify studies that investigated risk factors for major bleeding associated with DOAC treatment in patients diagnosed with VTE. Among the evaluated prognostic factors, quantitative pooling was possible only for age, which was reported in two studies; the remaining factors were summarized individually. Low-certainty evidence from a single study suggested a possible association between non-recommended dosing regimens and major bleeding. Evidence regarding declining adherence was also limited and of very low certainty. For age, very low-certainty evidence from the pooled analysis of two studies did not demonstrate a statistically significant association with major bleeding.

**Conclusion:**

The available evidence suggests possible associations between several prognostic factors and major bleeding in patients with VTE treated with DOACs; however, most findings are supported by low- or very low-certainty evidence and frequently by single studies. These findings should therefore be interpreted cautiously and require validation in larger, high-quality prospective studies.

## 1 Introduction

Venous thromboembolism (VTE), which includes pulmonary embolism (PE) and deep vein thrombosis (DVT), ranks as the third most common cardiovascular disease after myocardial infarction and stroke (1). Direct oral anticoagulants (DOACs) have become the preferred treatment for VTE, as recommended by the 2021 American College of Chest Physicians (ACCP) guidelines on antithrombotic therapy for VTE and the 2019 European Society of Cardiology (ESC) Guidelines for the Diagnosis and Management of Acute Pulmonary Embolism, offering advantages over traditional therapy with heparin and vitamin K antagonists (VKAs) (2, 3). DOACs, including dabigatran, rivaroxaban, apixaban, and edoxaban, have a shorter half-life and eliminate the need for routine coagulation monitoring, providing a more predictable response and allowing for flexible dosing (4).

Studies by Nick van Es and Lutsey PL have confirmed the favorable efficacy and safety of DOACs in VTE treatment compared to VKAs (4, 5). However, major bleeding (MB) remains a serious complication, with a significant increase in mortality within 30 days of occurrence (6). For instance, Agnelli et al. reported a 0.6% incidence of major bleeding in VTE patients treated with apixaban, while the Hokusai-VTE study found similar bleeding outcomes in patients treated with edoxaban versus VKAs (7, 8). For the initial and long-term treatment of pulmonary embolism, fixed-dose rivaroxaban regimens alone are non-inferior to conventional therapies and may improve the benefit-risk profile, with a significant difference observed for MB (9). Treatment with DOACs has been associated with a lower risk of major bleeding, intracranial hemorrhage, fatal bleeding, and clinically relevant non-major bleeding compared with conventional anticoagulant therapy (10). Despite these benefits, major bleeding remains an important complication of anticoagulant therapy, highlighting the need for improved assessment of factors associated with bleeding risk.

Previous studies have reported associations between several clinical characteristics and MB in anticoagulated patients, including female sex, concomitant antiplatelet use, low hemoglobin levels, and hypertension (11). Bortoluzzi et al. reported an association between higher HAS-BLED scores and increased bleeding risk (12). However, existing bleeding risk prediction models were largely developed or validated in populations receiving vitamin K antagonists or in broader anticoagulated populations, and their applicability to DOAC-treated patients with VTE remains uncertain. This systematic review and meta-analysis aimed to synthesize the available evidence on prognostic factors associated with major bleeding in patients receiving DOACs for VTE and to evaluate the certainty of evidence supporting these associations.

## 2 Method

This systematic review and meta-analysis were conducted in strict adherence to the guidelines set forth by the Preferred Reporting Items for Systematic Reviews and Meta-Analyses (PRISMA) (13). This methodological framework ensures transparency, reproducibility, and comprehensive reporting, which are essential for evaluating the rigor of systematic reviews. The protocol for this review was prospectively registered with PROSPERO (CRD 42023464518), a recognized database for registering systematic reviews, ensuring that the review process adhered to predefined criteria and was free from *post hoc* modifications.

### 2.1 Patient and public involvement

Patients and the public were not involved in the design, conduct, reporting, or dissemination of this systematic review and meta-analysis.

### 2.2 Search strategy

A systematic literature search was conducted in PubMed and Web of Science for studies published in English. The search was originally conducted in June 2023, consistent with the PROSPERO registration, and was re-run on September 18, 2026, using the identical search strategy, which yielded one additional eligible study (reference 24) that was added to the review. In addition, the reference lists of relevant articles were manually screened to identify potentially eligible studies that were not retrieved through the database search. A detailed description of the search strategy is provided in Supplementary Table S1. Study selection was performed independently by two reviewers who first screened titles and abstracts and then assessed full texts against the predefined eligibility criteria; disagreements were resolved by discussion or adjudication by a third reviewer. EndNote was used for reference management and duplicate removal.

### 2.3 Inclusion and/or exclusion criteria

Studies eligible for inclusion in this review were those that involved adult patients (aged ≥18 years) diagnosed with VTE who were treated with DOACs. The study had to compare patients who experienced MB during DOAC treatment with those who did not experience MB. The inclusion criteria were not restricted to any particular study design and were applicable to both observational studies (prospective and retrospective cohort, real-world observational, and registry studies) and randomized controlled trials (RCTs). The primary outcome of the study had to focus on identifying risk factors or predictors of MB events in VTE patients treated with DOACs, and it had to provide complete data on the relevant risk factors and outcomes. Studies were excluded if they involved patients treated with anticoagulants other than DOACs. Additionally, studies were excluded if they had incomplete data, including missing information on key risk factors or outcomes, or if they were in the design or recruitment phase with no available data. Studies where attempts to contact the corresponding author for additional data were unsuccessful, and the necessary data could not be obtained were also excluded. Studies conducted in pediatric populations (aged <18 years) were excluded.

### 2.4 Data extraction

For each included study, we extracted the first author, year of publication, study period, study design, sample size, patient age and sex distribution, VTE population, DOAC treatment, and number of major bleeding events where available. For prognostic factor studies, we extracted the reported prognostic factors and measures of association, including the odds ratio (*OR*), risk ratio (*RR*), hazard ratio (*HR*), or subdistribution hazard ratio (*sHR*), together with the corresponding 95% confidence interval (*CI*) and *P*-value where reported. For studies evaluating bleeding risk prediction scores, the names of the prediction scores and their reported effect estimates were also extracted. Major bleeding was defined according to the International Society on Thrombosis and Haemostasis (ISTH) criteria as: (1) symptomatic bleeding in a critical area or organ; (2) bleeding causing a decrease in hemoglobin level of at least 2 g/dL or requiring transfusion of at least two units of whole blood or red blood cells; or (3) fatal bleeding (14). Non-recommended dosing was defined as dosing outside the label-recommended (Summary of Product Characteristics) dose for VTE; in the included study by Chopard et al. (21), this specifically denoted subtherapeutic underdosing.

### 2.5 Quality assessment

#### 2.5.1 Risk of bias assessment

We assessed the risk of bias of prognostic factor studies using the Quality in Prognosis Studies (QUIPS) tool and evaluated studies of bleeding risk prediction models using the Prediction Study Risk of Bias Assessment Tool (PROBAST) (15–17).

#### 2.5.2 Certainty of evidence assessment

We evaluated the certainty of evidence for the association between each prognostic factor and major bleeding using the Grading of Recommendations Assessment, Development and Evaluation (GRADE) approach. The assessment considered risk of bias, indirectness, inconsistency, imprecision, and publication bias. Based on these domains, an overall certainty rating of high, moderate, low, or very low was assigned to each association, and evidence profiles were developed accordingly (18).

### 2.6 Statistical analysis

We standardized each risk factor through log transformation and unified the directionality of the predictors. Effect estimates were extracted as reported in the original studies. Adjusted effect estimates were used for quantitative synthesis when available to reduce the potential influence of measured confounding. Adjusted and unadjusted estimates were not pooled together for the same prognostic factor. Quantitative pooling across studies was undertaken only when studies evaluated the same or sufficiently comparable prognostic factors and provided effect estimates that could be harmonized. Before pooling, the definitions and directionality of prognostic factors were reviewed for clinical comparability. Most prognostic factors were reported by a single eligible study and were therefore summarized individually rather than pooled across studies. Because the included studies differed in patient populations, prognostic factor definitions, statistical models, and covariate adjustment, the degree of control for potential confounding was not uniform across studies, and residual confounding could not be excluded.

Because the included studies reported different measures of association, including odds ratios (*ORs*), risk ratios (*RRs*), and hazard ratios (*HRs)*, *ORs* were used as the common effect measure for quantitative synthesis. Reported *RRs* and *HRs* were converted to ORs using baseline risk information provided in the respective studies according to the published methods cited in this review (19, 20). This conversion was used to facilitate synthesis across studies reporting different effect measures; however, it relies on assumptions and may introduce approximation error. Major bleeding was uncommon in studies for which event incidence could be calculated (<10%), which may reduce, but does not eliminate, discrepancies among effect measures. Sensitivity analyses retaining the original effect measures were considered. However, these analyses were not performed because only a small number of studies contributed to each prognostic factor, and the number of studies reporting the same original effect measure was insufficient for separate measure-specific meta-analyses. Directly pooling ORs, RRs, and HRs without harmonization was considered methodologically inappropriate because these measures are not directly equivalent. Therefore, where quantitative synthesis was feasible, effect estimates were harmonized to ORs, and the resulting pooled estimates were interpreted cautiously. Meta-analyses were performed using Review Manager 5.3 software, employing fixed-effect or random-effects models based on the level of statistical heterogeneity. The Chi-Square test assessed heterogeneity among study results, with *P* ≥ 0.1 and *I^2^* ≤ 50% indicating acceptable homogeneity, thus justifying the use of a fixed-effect model. Conversely, *P* < 0.1 or *I^2^* > 50% signified substantial heterogeneity, prompting the application of a random-effects model. The statistical indicators were the *OR* and 95% *CI*. Given the small number of studies contributing to individual prognostic factors, estimates of statistical heterogeneity were interpreted cautiously.

## 3 Results

### 3.1 The characteristics of included studies

Our initial search yielded 4,105 citations, from which 52 studies were selected for full-text assessment. Ultimately, 7 articles were identified as meeting the inclusion criteria and were included in this study. Figure 1 presents the PRISMA flowchart, which visually outlines the selection process. Table 1 summarizes the main characteristics of the included studies, while Supplementary Table S2 provides more detailed study characteristics and outcome information. All included studies provided sufficient statistical information for effect size estimation. However, most prognostic factors were evaluated in only one study, and quantitative pooling across studies was therefore possible for only a limited number of factors.

**Figure 1:**
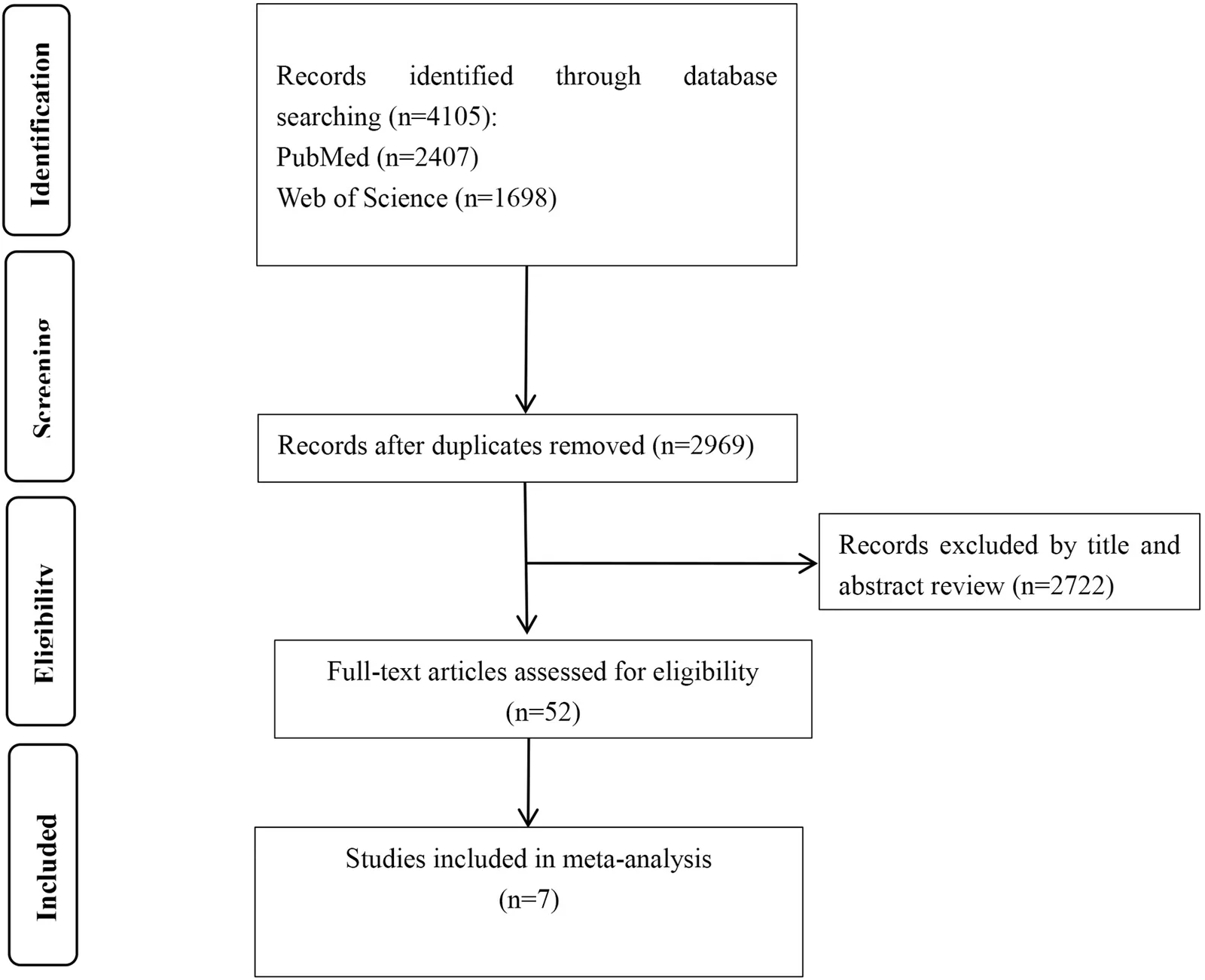
PRISMA flow diagram of study selection.

**Table 1:** Characteristics of the seven included studies.

First author, year	Study design	Population	Age, years	Sample size	Sex, male, *n* (%)	No. of MB	DOAC agent
Vedovati MC, 2020(10)	Prospective multicenter cohort	VTE	66.7 (16.2)	1,034	499 (51.7)	25	DOACs
Chopard R, 2018(21)	Prospective multicenter cohort	Acute pulmonary embolism	63.6 (17.9)	656	308 (46.9)	11	Rivaroxaban; Apixaban
Jin MC, 2021(22)	Retrospective cohort	VTE	63.1 (15.7)	225,559 (Apixaban 34,201; Rivaroxaban 46,007)	6,924 (43.9) Apixaban; 11,435 (43.9) Rivaroxaban	NR	Apixaban; Rivaroxaban
Park H, 2023(23)	Retrospective cohort	VTE (Medicare beneficiaries)	72.6 (11.3)	6,597	3,877 (41.2)	144	DOACs
Kobayashi S, 2025(24)	Retrospective cohort	VTE	67.7 (15.5)	4,959 (DOAC 3,940)	2,042 (41.2)	454	DOACs (edoxaban 53%)
Nakamura M, 2020(25)	Prospective observational (real-world)	VTE (Japan)	68.0 (14.3)	1,702	1,026 (39.7)	45	Edoxaban
Wallvik N, 2021(26)	Retrospective registry	VTE	69.4 (56.8–78.5)^¶^	18,219	9,544 (52.4)	938	DOACs (NOAC)

DOAC, direct oral anticoagulant; MB, major bleeding; NR, not reported; SD, standard deviation; VTE, venous thromboembolism. Values for age are presented as mean (SD) unless otherwise indicated. ^¶^Median (interquartile range).

Among the seven included studies, five were cohort studies, including two prospective multicenter cohort studies and three retrospective cohort studies (10, 21–24). Additionally, one study was a prospective, observational real-world study, and one was a retrospective registry study (25, 26). All seven studies focused on populations of patients with VTE, although one study specifically concentrated on patients with acute pulmonary embolism (21). The majority of patients were aged between 50 and 80 years, with a slight predominance of females over males. On average, male participants represented 46.0% of the study population.

### 3.2 Risk of bias assessment

The risk of bias was identified as high across all included studies, with each study exhibiting bias in at least one domain or item (Supplementary Table S3). Of the 7 studies included, 4 were retrospective, a design feature that may have introduced classification bias, particularly in terms of outcome measurement and data interpretation (22–24, 26). Supplementary Tables S3, S4 provide detailed assessments of the risk-of-bias domains and the certainty of evidence, respectively.

### 3.3 Prognostic factors associated with major bleeding during DOAC treatment for VTE

Eighteen candidate variables were evaluated across the seven studies included in this review, comprising individual prognostic factors and composite bleeding risk prediction scores. Of these 18 variables, only age was evaluated in more than one eligible study and was therefore the only factor for which a pooled meta-analysis was performed. The remaining 17 variables were each reported by a single study and were summarized individually without quantitative pooling. Supplementary Table S4 summarizes the certainty of evidence for the evaluated factors. Supplementary Figures 1–18 present the study-specific or pooled effect estimates, as applicable.

### 3.4 Demographic factors

Very low-certainty evidence from one study suggested that female sex may be associated with an increased risk of MB (*OR*, 1.66; 95% *CI*, 1.25–2.21) (22). For age, very low-certainty evidence from two studies did not demonstrate a statistically significant association with MB (*OR*, 1.19; 95% *CI*, 0.89–1.59) (22, 26).

### 3.5 Bleeding risk prediction scores

One prospective cohort study evaluated the ATRIA, ORBIT, and VTE-BLEED scores for predicting MB in patients with VTE treated with DOACs (10). Very low-certainty evidence suggested possible associations between the Anticoagulation and Risk Factors in Atrial Fibrillation (ATRIA) and Outcomes Registry for Better Informed Treatment (ORBIT) scores and MB risk. The ATRIA score was associated with an *OR* of 3.04 (95% *CI*, 1.38–6.71), while the ORBIT score had an *OR* of 3.62 (95% *CI*, 1.65–7.94) (10). Low-certainty evidence suggested a possible association between the VTE-BLEED score and MB (*OR*, 16.12; 95% *CI*, 2.18–119.24) (10).

### 3.6 Medical illness and patient history factors

Several underlying medical conditions showed possible associations with MB, although the certainty of evidence was very low. These included complicated hypertension (*OR*, 1.94; 95% *CI*, 1.13–3.31), hypertension (*OR*, 2.11; 95% *CI*, 1.15–3.86), and a history of early major bleeding (*OR*, 1.58; 95% *CI*, 1.09–2.30) (22, 25, 26). Additionally, prior stroke (*OR*, 1.28; 95% *CI*, 1.03–1.58), transient ischemic attack (TIA) (*OR*, 1.33; 95% *CI*, 1.01–1.76), cerebrovascular disease (*OR*, 2.78; 95% *CI*, 1.48–5.20), cancer (*OR*, 3.32; 95% *CI*, 1.84–6.01), chronic obstructive pulmonary disease (COPD) (*OR*, 1.29; 95% *CI,* 1.04–1.60), and low body weight (BW) (≤60 kg)(>60 kg vs ≤60 kg: *OR*, 0.73; 95% *CI*, 0.60–0.89) were also reported to be associated with MB in the included studies (22, 24–26).

### 3.7 Medication factors

The certainty of evidence for medication-related factors ranged from low to very low. Very low-certainty evidence suggested that prior prescription of antiplatelet therapy was associated with an increased risk of MB (*OR*, 1.59; 95% *CI*, 1.25–2.02), as was declining adherence to DOACs (*OR*, 1.80; 95% *CI*, 1.07–3.03) (22, 23). Low-certainty evidence from one prospective cohort study suggested a possible association between non-recommended dosing regimens and MB (*OR*, 9.92; 95% *CI*, 1.80–54.71) (21). Finally, prior warfarin therapy was associated with a lower risk of MB (*OR*, 0.67; 95% *CI*, 0.58–0.78), although the certainty of evidence was very low (26).

## 4 Discussion

This systematic review synthesized the available evidence on prognostic factors associated with MB in patients with VTE treated with DOACs. Although several possible associations were identified, the evidence base was limited, with only seven observational studies and predominantly low or very low certainty of evidence. Therefore, the findings should be interpreted primarily as hypothesis-generating and may help inform future research and refinement of bleeding risk assessment strategies rather than directly guide clinical decision-making.

Several clinical characteristics showed statistically significant associations with MB in individual included studies, including female sex, hypertension, complicated hypertension, a history of major bleeding, prior stroke, TIA, cerebrovascular disease, cancer, COPD, and low BW (≤60 kg). However, the certainty of evidence supporting most of these associations was very low, and many were evaluated in only one study. These findings therefore require validation before they can be incorporated into clinical decision-making or used to modify existing bleeding risk prediction models (27). The management of underlying conditions such as hypertension and cerebrovascular disease may help reduce the incidence of MB by preventing secondary complications like stroke and TIA, which are known to exacerbate bleeding risks (28). Furthermore, controlling comorbidities such as COPD could potentially improve patient outcomes by minimizing bleeding-related events (26). Our findings regarding the associations of hypertension and cancer with MB differ from those of Vedovati et al., who reported no significant relationship between these factors and MB in VTE patients treated with DOACs (10). This discrepancy may stem from differences in study design, sample size, and patient characteristics. Given that our study had a smaller sample size, the results should be interpreted with caution, highlighting the need for larger, more robust studies to validate these possible associations. We also observed very low-certainty evidence suggesting a possible association between MB risk and sex, particularly in relation to abnormal uterine bleeding in women treated with DOACs. Previous studies have shown that women on rivaroxaban or apixaban are at a higher risk of severe uterine bleeding compared to warfarin (29–31). These observations warrant further investigation of sex-specific bleeding risks in patients receiving DOACs. Further research is necessary to better understand the mechanisms behind these sex-specific bleeding risks and to refine treatment guidelines for this population.

Based on very low-certainty evidence, the pooled analysis did not demonstrate a statistically significant association between age and MB risk. However, the absence of statistical significance should not be interpreted as evidence that age has no effect, particularly given the limited number of included studies. Previous studies have reported inconsistent findings; for example, Ruigómez et al. observed an increase in bleeding risk with advancing age among rivaroxaban users (32). These findings suggest that the relationship between age and bleeding risk remains uncertain and may be influenced by comorbidities, polypharmacy, and DOAC-specific treatment characteristics.

The ATRIA, ORBIT, and VTE-BLEED scores are composite bleeding risk prediction models rather than individual prognostic factors and should therefore be considered separately. In the single prospective cohort study that evaluated these scores in patients with VTE receiving DOACs, higher-risk categories defined by ATRIA, ORBIT, and VTE-BLEED were associated with an increased risk of major bleeding (10). However, these findings were derived from only one study, and the certainty of evidence was low or very low. Therefore, the comparative predictive performance and clinical utility of these scores in DOAC-treated patients with VTE remain uncertain. Further external validation in independent cohorts is required before these models can be reliably used to guide clinical decision-making in this population.

Regarding medication adherence, one included study reported an association between progressively declining adherence to DOAC therapy and an increased risk of major bleeding (23). However, this finding was derived from a single observational study with very low-certainty evidence. The direction and mechanisms underlying this association remain uncertain, and residual confounding or changes in medication-taking behavior related to bleeding events cannot be excluded. Therefore, this finding should be considered exploratory and requires confirmation in prospective studies. Similarly, one included study reported that prior warfarin therapy was associated with a lower subsequent risk of major bleeding during DOAC treatment (26). Given the observational design and very low certainty of evidence, this association should not be interpreted as demonstrating a protective effect of prior warfarin exposure. Differences in patient characteristics, treatment history, and residual confounding may have contributed to the observed association. Further studies are needed to determine whether this finding is reproducible and clinically relevant.

Although our meta-analysis pooled data across all DOAC subgroups, recent evidence has suggested clinically relevant differences in bleeding risk among individual agents. One international phase 4 prospective, randomized, open-label, blinded-endpoint trial enrolled 2,760 patients with acute symptomatic pulmonary embolism or proximal deep vein thrombosis to compare bleeding risks between apixaban and rivaroxaban (33). At the 3-month follow-up, apixaban was associated with a substantially lower risk of clinically relevant bleeding (*RR*, 0.46; *P* < 0.001) (33). The corresponding editorial noted that anticoagulant selection is no longer an arbitrary decision for most patients with acute VTE (34). A FAERS database study comparing bleeding profiles of the four commonly used DOACs reported the highest proportional reporting ratio (PRR) for major bleeding with edoxaban (14.1), followed by dabigatran (4.0), apixaban (3.53), and rivaroxaban (2.11). Another meta-analysis evaluating comparative efficacy and safety of different DOACs in VTE patients also reported that apixaban carried a lower bleeding risk relative to rivaroxaban (35). Unfortunately, nearly all primary studies included in our analysis failed to report disaggregated effect sizes stratified by individual DOAC agent, which prevented us from conducting robust drug-specific subgroup meta-analyses. Therefore, pooling different DOAC agents may have obscured agent-specific differences in bleeding risk and represents an important limitation of the present analysis. Further head-to-head randomized trials and large-scale real-world cohort studies that adjust for potential confounders, including treatment indication and administered dose, are required to delineate agent-specific bleeding risk patterns among patients with VTE. The COBRRA trial was not eligible for inclusion in the present review because, although our inclusion criteria did not exclude RCTs, it compared bleeding outcomes between two treatment arms (apixaban versus rivaroxaban) rather than evaluating patient-level prognostic factors associated with major bleeding within a DOAC-treated cohort. It is therefore cited here only for context on agent-specific bleeding differences.

The available evidence suggested a possible association between non-recommended DOAC dosing and an increased risk of major bleeding (*OR*, 9.92), although this finding was derived from only one eligible prospective multicenter cohort study by Chopard et al. The study enrolled 656 patients with acute pulmonary embolism receiving DOACs after discharge, among whom 4.3% received subtherapeutic non-recommended doses. The composite endpoint of all-cause death, major bleeding, or recurrent VTE occurred in 25.0% of patients with non-recommended dosage versus 6.1% in the standard-dose group (*RR*, 3.19; *P* < 0.001). The disparity was predominantly driven by major bleeding events, with an incidence of 7.1% in the non-recommended dosage group and 1.4% in the standard-dose group (*P* = 0.008) (21). Notably, evidence derived from a single study limits the robustness and generalizability of this finding, which should therefore be interpreted as exploratory. More high-quality original studies are required to validate the association between non-recommended dosing and DOAC-related major bleeding among VTE patients. Nevertheless, dosing appropriateness remains clinically relevant because inappropriate dosing may affect both bleeding and thromboembolic outcomes. Unlike warfarin, DOACs do not require routine coagulation monitoring, making dose errors more difficult to identify in routine practice. Subtherapeutic drug exposure has been associated with an increased risk of recurrent thromboembolism without a clear bleeding-related safety benefit (36–39). These findings support careful adherence to label-recommended dosing and individualized dose adjustment according to renal function, BW and clinical characteristics. However, the incremental predictive value of dosing appropriateness should be evaluated in future prospective studies before it is incorporated into bleeding prediction models. Of note, “non-recommended dosing” as used in this review is restricted to dosing below the label-recommended dose for VTE (subtherapeutic underdosing), consistent with the definition used in the included study by Chopard et al., and should not be conflated with the broader concept of “off-label” dosing, which may also include overdosing.

However, this study has several limitations. Data were sourced from PubMed and Web of Science, which may not capture all relevant studies. Although reference lists were manually screened, relevant studies indexed in other databases may still have been missed. Additionally, only seven observational studies, four of which were retrospective, were included. Most prognostic factors were evaluated in only one study, and the certainty of evidence was predominantly low or very low. Moreover, the included studies differed in patient populations, definitions of prognostic factors, statistical models, and covariate adjustment, which may have introduced clinical and methodological heterogeneity and limited control for confounding. Consequently, these limitations may have reduced the reliability and generalizability of the effect estimates. Pooling different DOAC agents may also have obscured agent-specific differences in bleeding risk because drug-specific effect estimates were generally unavailable. In addition, the two studies contributing to the pooled analysis of age reported different effect measures, specifically a subdistribution *HR* and an *HR*, which were harmonized to ORs for quantitative synthesis. Although major bleeding was uncommon in studies for which event incidence could be calculated, the conversions rely on assumptions and may introduce approximation error, particularly when baseline risks and follow-up periods differ across studies. Sensitivity analyses retaining the original effect measures could not be meaningfully performed because too few studies contributed to individual prognostic factors to permit separate pooling according to the original effect measure. Therefore, the pooled estimate should be interpreted cautiously.

## 5 Conclusion

The available evidence suggests possible associations between several prognostic factors and major bleeding in patients with VTE treated with DOACs, but most findings are based on low- or very low-certainty evidence and often on single observational studies. These findings should therefore be interpreted cautiously and require validation in larger, high-quality prospective studies.

## Data Availability

The original contributions presented in the study are included in the article/Supplementary Material, further inquiries can be directed to the corresponding author.
