# Supplementary material for: Risk factors of major bleeding associated with direct oral anticoagulant treatment in patients with venous thromboembolism: a systematic review and meta-analysis
Source: Front Med (Lausanne). 2026 Oct 15;13:1869818. doi: 10.3389/fmed.2026.1869818 (PMC13716557; doi:10.3389/fmed.2026.1869818)
Supplement: Supplementary file 1 [file Table1.docx]

**sFigure 1. Forest plot of risk factors for major bleeding with sex**

**sFigure 2. Forest plot of risk factors for major bleeding with older age**

**sFigure 3.** **Forest plot of the ATRIA bleeding risk prediction score for major bleeding**

**sFigure 4.** **Forest plot of the ORBIT bleeding risk prediction score for major bleeding**

**sFigure 5.** **Forest plot of the VTE-BLEED bleeding risk prediction score for major bleeding**

**sFigure 6. Forest plot of risk factors for major bleeding with complicated hypertension**

**sFigure 7. Forest plot of risk factors for major bleeding with hypertension**

**sFigure 8. Forest plot of risk factors for major bleeding with early major bleeding**

**sFigure 9. Forest plot of risk factors for major bleeding with previous stroke**

**sFigure 10. Forest plot of risk factors for major bleeding with transient ischemic attack**

**sFigure 11. Forest plot of risk factors for major bleeding with cerebrovascular disease**

**sFigure 12. Forest plot of risk factors for major bleeding with cancer**

**sFigure 13. Forest plot of risk factors for major bleeding with COPD**

**sFigure 14. Forest plot of risk factors for major bleeding with prior prescription of antiplatelet therapy**

**sFigure 15. Forest plot of risk factors for major bleeding with decline in adherence to DOACs**

**sFigure 16. Forest plot of risk factors for major bleeding with non-recommended dosage**

**sFigure 17. Forest plot of risk factors for major bleeding withprior warfarin therapy**

**sFigure 18. Forest plot of risk factors for major bleeding with low BW (≤60kg)**
