# Supplementary material for: Risk factors of major bleeding associated with direct oral anticoagulant treatment in patients with venous thromboembolism: a systematic review and meta-analysis
Source: Front Med (Lausanne). 2026 Oct 15;13:1869818. doi: 10.3389/fmed.2026.1869818 (PMC13716557; doi:10.3389/fmed.2026.1869818)
Supplement: Supplementary file 2 [file Table2.docx]

**Supplementary Table1. Search strategy**

| **1.PubMed** | |
| --- | --- |
| #1 | Dabigatran[MeSH] OR Rivaroxaban[MeSH] OR Factor Xa Inhibitors[MeSH] |
| #2 | dabigatran[Title/Abstract] OR Pradaxa[Title/Abstract] OR rivaroxaban[Title/Abstract] OR Xarelto[Title/Abstract] OR apixaban[Title/Abstract] OR Eliquis[Title/Abstract] OR edoxaban[Title/Abstract] OR Savaysa[Title/Abstract] OR betrixaban[Title/Abstract] OR "factor xa inhibitor*"[Title/Abstract] OR "factor 10a inhibitor*"[Title/Abstract] OR "factor iia inhibitor*"[Title/Abstract] OR "direct thrombin inhibitor*"[Title/Abstract] OR "non vitamin k antagonists oral anticoagulant*"[Title/Abstract] OR "non vitamin k antagonist*"[Title/Abstract] OR noac*[Title/Abstract] OR "direct oral anticoagulant*"[Title/Abstract] OR doac*[Title/Abstract] OR "novel oral anticoagulant*"[Title/Abstract] OR "new oral anticoagulant*"[Title/Abstract] OR "new orally active anticoagulant*"[Title/Abstract] |
| #3 | Venous Thromboembolism[Mesh] OR Venous Thrombosis[Mesh] OR Pulmonary Embolism[Mesh] |
| #4 | Vein Thromboembolism[Title/Abstract] OR Vein Thrombosis[Title/Abstract] OR venous thromboem*[Title/Abstract] OR venous thrombos*[Title/Abstract] OR deep vein thrombos*[Title/Abstract] OR deep venous thrombos*[Title/Abstract] OR phlebothrombos*[Title/Abstract] OR pulmonary embolism[Title/Abstract] OR pulmonary thromboembolism[Title/Abstract] OR lung embolism[Title/Abstract] OR VTE[Title/Abstract] OR DVT[Title/Abstract] OR PE[Title/Abstract] |
| #5 | Hemorrhage[MeSH Terms] OR Bleeding[MeSH Terms] |
| #6 | Hemorrhage[Title/Abstract] OR Bleeding[Title/Abstract] |
| #7 | risk factors[MeSH Terms] OR Causality[MeSH Terms] OR probability[MeSH Terms] OR Prognosis[MeSH Terms] OR risk assessment[MeSH Terms] |
| #8 | "risk factor*"[Title/Abstract] OR Causality[Title/Abstract] OR cause*[Title/Abstract] OR probability[Title/Abstract] OR Prognosis[Title/Abstract] OR "risk assessment"[Title/Abstract] OR "Prediction model"[Title/Abstract] OR "Predicting"[Title/Abstract] OR "Predicting risk"[Title/Abstract] OR "Risk prediction"[Title/Abstract] OR "Risk Score"[Title/Abstract] OR "Risk Factor Score"[Title/Abstract] OR "Relative Risk"[Title/Abstract] |
| #9 | #1 OR #2 |
| #10 | #3 OR #4 |
| #11 | #5 OR #6 |
| #12 | #7 OR #8 |
| #13 | #9 AND #10 AND #11 AND #12 |
| **2.Web of science** | |
| #1 | TS= Rivaroxaban OR TS= Xarelto OR TS= dabigatran OR TS= Pradaxa OR TS= apixaban OR TS= Eliquis OR TS= edoxaban OR TS= Savaysa OR TS= betrixaban OR TS= "factor Xa inhibitor*" OR TS= "factor 10a inhibitor*" OR TS= "factor IIa inhibitor*" OR TS= "direct thrombin inhibitor*" OR TS= "non-vitamin K antagonists oral anticoagulant*" OR TS= "non-vitamin K antagonist*" OR TS= NOAC* OR TS= "direct oral anticoagulant*" OR TS= DOAC* OR TS= "novel oral anticoagulant*" OR TS= "new oral anticoagulant*" OR TS= "new orally active anticoagulant*" |
| #2 | TS= "Venous Thromboembolism" OR TS= "Venous Thrombosis" OR TS= "Pulmonary Embolism" OR TS= "Vein Thromboembolism" OR TS= "Vein Thrombosis" OR TS= "venous thromboem*" OR TS= "venous thrombos*" OR TS= "deep vein thrombos*" OR TS= "deep venous thrombos*" OR TS= "phlebothrombos*" OR TS= "pulmonary embolism" OR TS= "pulmonary thromboembolism" OR TS= "lung embolism" OR TS= "VTE" OR TS= "DVT" OR TS= "PE" |
| #3 | TS= Hemorrhage OR TS= Bleeding |
| #4 | TS="risk factor*" OR TS= Causality OR TS= cause* OR TS= probability OR TS= Prognosis OR TS= "risk assessment" OR TS= "Prediction model" OR TS= Predicting OR TS= "Predicting risk" OR TS= "Risk prediction" OR TS= "Risk Score" OR TS= "Risk Factor Score" OR TS= "Relative Risk" |
| #5 | #1 AND #2 AND #3 AND #4 |

**Supplementary Table 2. Characteristics of the included studies**

| **First author, year** | **Mean age, y (SD）/ Range** | **Sex, male, n (%)** | **Drug** | **Population** | **Sample size** | **time** | **Study design** | **Number**  **of MB** | **Risk factor** |
| --- | --- | --- | --- | --- | --- | --- | --- | --- | --- |
|  |  |  |  |  |  |  |  |  |  |
| Chopard, R., et al. (2018)^21^ | 63.6 (17.9) | Male  308 (46.9） | Rivaroxaba Apixaban | acute pulmonary embolism | 656 | September 1, 2012 to April 30, 2017 | Prospective, multicenter cohort study | 11 | Non-recommended dose (*HR*=9.37, 95% *CI*:1.80–54.7, *P* =0.008); |
| Vedovati, M. C., et al. (2020)^10^ | 66.7(16.2) (19-99） | Male  499(51.7) | DOAC | VTE | 1034 | March 2014, to November 2017. | multicenter prospective cohort study | 25 | the ATRIA score (*HR*=3.05, 95% *CI*：1.38 to 6.71, *P* =0.006)  the ORBITscore (*HR* =3.62, 95% *CI* ：1.65to 7.94, *P* =0.001);  theVTE-BLEED score (HR=16.11, 95%CI： 2.18to 119.09, *P* =0.006). |
| Nakamura, M., et al. (2020)^25^ | 68.0(14.3) | Male 1026(39.7) | Edoxaban | VTE | 1702 | 1 February 1, 2015 to July 31, 2017 | prospective, observational real-world study | 45 | Baseline comorbidity:hypertension *HR*=2.109， 95% *CI*: (1.154-3.854)，*P* = 0.0154  Baseline comorbidity: cancer *HR*=3.324， 95% *CI*: (1.839-6.006)，*P* <0.0001 |

**Supplementary Table 2. Characteristics of the included studies (Continued)**

| **First author, year** | **Mean age, y (SD）/ Range** | **Sex, male, n (%)** | **Drug** | **Population** | **Sample size** | **time** | **Study design** | **Number**  **of MB** | **Risk factor** |
| --- | --- | --- | --- | --- | --- | --- | --- | --- | --- |
| Jin, M. C., et al. (2021)^22^ | 63.1(15.7) | Male  Apixaban 6924 (43.9)  Rivaroxaban 11435 (43.9) | Aspirin Rivaroxaban | VTE | 225559  Apixaban 34201  Rivaroxaban46007 | 2003 to 2019 | Retrospective cohort study | MB; no data | Apixaban  older age (*sHR* =1.025, 95% *CI* =1.011–1.039),  female sex (*sHR* =1.662, 95% *CI* =1.252–2.207),  prior prescription antiplatelet therapy (*sHR* =1.591, 95% *CI* =1.130–2.241),  complicated hypertension (*sHR* =1.936, 95% *CI* =1.134–3.307).  DOAC  Intracerebral hemorrhage Cerebrovascular disease (*HR* =2.778, 95% *CI* =1.485–5.197,*P*=0.001). |
| Wallvik, N., et al. (2021)^26^ | 69.4 (56.8–78.5) | Male  9544(52.4) | NOAC | VTE | 18219 | 2012 to 2017 | Retrospective register-based study | 938 | age (normalized *HR* 1.38, *CI* 1.27–1.50),  earlier major bleeding (*HR* 1.58, *Cl* 1.09–2.30),  COPD (*HR* 1.28, *CI* 1.04–1.60)  previous stroke (*HR* 1.28, *Cl* 1.03–1.58)  transient ischemic attack (TIA) (*HR* 1.33, *Cl* 1.01–1.76).  Prior warfarin treatment was protective (*HR* 0.67, *CI* 0.58–0.78). |
| Park, H., et al. (2023)^23^ | 72.6(11.3) | Male  3877(41.2) | DOAC | VTE | 6597 | 2014 to 2018 | Retrospective cohort study | 144 | gradually declining DOAC adherence (*HR* =1.80; 95% *CI* = 1.07-3.03), *P* <0.05 |

**Supplementary Table 2. Characteristics of the included studies (Continued)**

| **First author, year** | **Mean age, y (SD）/ Range** | **Sex, male, n (%)** | **Drug** | **Population** | **Sample size** | **time** | **Study design** | **Number**  **of MB** | **Risk factor** |
| --- | --- | --- | --- | --- | --- | --- | --- | --- | --- |
| Kobayashi, S., et al. (2025)^24^ | 67.7 (15.5) | Male 2042 (41.2) | DOAC (Edoxaban 53%) | VTE | 4959 (DOAC 3940) | 2015 to 2020 (5-year follow-up) | Retrospective cohort study | MB 454 (Low BW 296 / Non-low BW 158) | Low body weight (≤60kg): DOAC-only adjusted HR 1.31 (95% *CI* 1.004–1.71, *P*=0.047); entire cohort HR 1.43 (95% *CI* 1.15–1.77, *P*=0.001) |

**Supplementary Table 3. Risk of bias assessments using QUIPS for prognostic factor studies**

| **Author** | **Year** | **Study**  **partici-pation** | **Study**  **attrition** | **Prognostic**  **factor**  **measurem-**  **ent** | **Outcome**  **measure-ment** | **Study**  **confound-ing** | **Statistical**  **analysis and**  **reporting** |
| --- | --- | --- | --- | --- | --- | --- | --- |
| Chopard, R., et al. (2018)^21^ | 2018 | low | low | low | low | low | moderate |
| Jin, M. C., et al. (2021)^22^ | 2021 | low | moderate | low | low | low | low |
| Nakamura, M., et al. (2020)^25^ | 2020 | low | moderate | low | low | low | low |
| Park, H., et al. (2023)^23^ | 2023 | low | low | low | low | low | low |
| Wallvik, N., et al. (2021)^26^ | 2021 | low | low | low | low | low | low |
| Vedovati, M. C., et al. (2020)^10^ | 2020 | low | low | low | low | low | low |
| Kobayashi, S., et al. (2025)^24^ | 2025 | low | low | low | low | low | moderate |

**Supplementary Table 4. Evidence profile for bleeding-related prognostic factors**

| **No. of**  **studies** | **Certainty assessment domains** | | | | | | **Overall certainty in the evidence about this prognostic factor** | **Relative effect**  **(*OR*, 95% *CI*)** |
| --- | --- | --- | --- | --- | --- | --- | --- | --- |
|  | **Study design** | **Risk of bias** | **Indirect** | **Inconsistent** | **Imprecise** | **Publication bias** |  |  |
| Non-recommended dose ^21^ | | | | | | | | |
| 1 | Observational | Serious | Not serious | Not serious | Serious | Undetected | ⊕⊕⊙⊙  LOW | 9.92, 1.8-54.71 |
| Older age ^22,26^ | | | | | | | | |
| 2 | Observational | Serious | Serious | Serious | Not serious | Undetected | ⊕⊙⊙⊙ VERY LOW | 1.19, 0.89-1.59 |
| Female sex ^22^ | | | | | | | | |
| 1 | Observational | Serious | Not serious | Not serious | Not serious | Undetected | ⊕⊙⊙⊙  VERY LOW | 1.66, 1.25-2.21 |
| Prior prescription antiplatelet therapy ^22^ | | | | | | | | |
| 1 | Observational | Serious | Not serious | Not serious | Not serious | Undetected | ⊕⊙⊙⊙  VERY LOW | 1.59, 1.25-2.02 |
| Complicated hypertension ^22^ | | | | | | | | |
| 1 | Observational | Serious | Not serious | Not serious | Not serious | Undetected | ⊕⊙⊙⊙  VERY LOW | 1.94, 1.13-3.31 |
| Cerebrovascular disease ^22^ | | | | | | | | |
| 1 | Observational | Serious | Not serious | Not serious | Not serious | Undetected | ⊕⊙⊙⊙  VERY LOW | 2.78, 1.48-5.20 |
| Hypertension ^25^ | | | | | | | | |
| 1 | Observational | Serious | Not serious | Not serious | Not serious | Undetected | ⊕⊙⊙⊙  VERY LOW | 2.11, 1.15-3.86 |
| Cancer ^25^ | | | | | | | | |
| 1 | Observational | Serious | Not serious | Not serious | Not serious | Undetected | ⊕⊙⊙⊙  VERY LOW | 3.32, 1.84-6.01 |
| Gradually declining DOAC adherence ^23^ | | | | | | | | |
| 1 | Observational | Serious | Not serious | Not serious | Not serious | Undetected | ⊕⊙⊙⊙  VERY LOW | 1.80, 1.07-3.03 |
| Earlier major bleeding ^26^ | | | | | | | | |
| 1 | Observational | Serious | Not serious | Not serious | Not serious | Undetected | ⊕⊙⊙⊙  VERY LOW | 1.58, 1.09-2.30 |
| COPD ^26^ | | | | | | | | |
| 1 | Observational | Serious | Not serious | Not serious | Not serious | Undetected | ⊕⊙⊙⊙  VERY LOW | 1.29, 1.04-1.60 |
| Previous stroke ^26^ | | | | | | | | |
| 1 | Observational | Serious | Not serious | Not serious | Not serious | Undetected | ⊕⊙⊙⊙  VERY LOW | 1.28, 1.03-1.58 |
| Transient ischemic attack (TIA) ^26^ | | | | | | | | |
| 1 | Observational | Serious | Not serious | Not serious | Not serious | Undetected | ⊕⊙⊙⊙  VERY LOW | 1.33, 1.01-1.76 |
| Prior warfarin treatment ^26^ | | | | | | | | |
| 1 | Observational | Serious | Not serious | Not serious | Not serious | Undetected | ⊕⊙⊙⊙  VERY LOW | 0.67, 0.58-0.78 |
| the ATRIA score ^10^ | | | | | | | | |
| 1 | Observational | Serious | Not serious | Not serious | Not serious | Undetected | ⊕⊙⊙⊙  VERY LOW | 3.04, 1.38-6.71 |
| the ORBITscore ^10^ | | | | | | | | |
| 1 | Observational | Serious | Not serious | Not serious | Not serious | Undetected | ⊕⊙⊙⊙  VERY LOW | 3.62, 1.65-7.94 |
| The VTE-BLEED score ^10^ | | | | | | | | |
| 1 | Observational | Serious | Not serious | Not serious | Serious | Undetected | ⊕⊕⊙⊙  LOW | 16.12, 2.18-119.24 |
| Low body weight (Kobayashi, S., et al.) ^24^ | | | | | | | | |
| 1 | Observational | Serious | Not serious | Not serious | Serious | Undetected | ⊕⊙⊙⊙ / VERY LOW | HR, 1.31 (95% CI, 1.004-1.71) |

**GRADE Working Group grades of evidence**

High certainty: we are very confident that the true effect lies close to that of the estimate of the effect.

Moderate certainty: we are moderately confident in the effect estimate: the true effect is likely to be close to the estimate of the effect, but there is a possibility that it is substantially different.

Low certainty: our confidence in the effect estimate is limited: the true effect may be substantially different from the estimate of the effect.

Very low certainty: we have very little confidence in the effect estimate: the true effect is likely to be substantially different from the estimate of effect.

**Explanations**

a. Risk of bias: The retrospective enrollment of patients may have introduced classification bias, certainty in evidence was downgraded for risk of bias.

b. Certainty in evidence was downgraded for high heterogeneity.

c. Imprecise: Certainty in evidence was downgraded for imprecision, given that the 95% CI suggests that there may be no association.
